# Butyl­bis­(diphenyl­glyoximato)(pyridine-κ*N*)­cobalt(III)^1^

**DOI:** 10.1107/S1600536812000153

**Published:** 2012-01-07

**Authors:** Sarvendra Kumar, Suresh Thapa

**Affiliations:** a DQIAQF/INQUIMAE, Universidad de Buenos Aires, Ciudad Universitaria, Pab. II, p. 3, EHA1428 Buenos Aires, Argentina; b Faculty of Science and Technology, Purbanchal University, Biratnagar, Nepal

## Abstract

In the title compound, [Co(C_4_H_9_)(C_14_H_11_N_2_O_2_)_2_(C_5_H_5_N)], the Co^III^ atom is coordinated by a butyl group, a nitro­gen-bonded pyridine and two *N*,*N*′-bidentate diphenyl­glyoximate ligands in a distorted octa­hedral geometry. The crystal structure features two short O—H⋯O bridges between the two chelating anions, with O⋯O distances less than 2.5 Å.

## Related literature

For background to the chemistry of cobaloximes, see: Schrauzer (1968 ▶); Zangrando *et al.* (2003 ▶); Brown (2006 ▶); Randaccio (1999 ▶). For related structues, see: Kumar & Gupta (2011 ▶); Mandal & Gupta (2005 ▶, 2007 ▶).
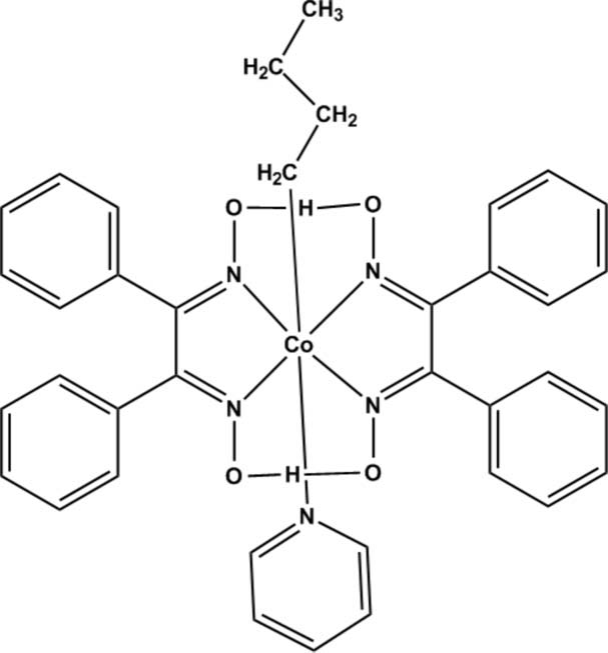


## Experimental

### 

#### Crystal data

[Co(C_4_H_9_)(C_14_H_11_N_2_O_2_)_2_(C_5_H_5_N)]
*M* *_r_* = 673.64
Monoclinic, 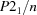

*a* = 11.863 (4) Å
*b* = 19.520 (7) Å
*c* = 14.402 (5) Å
β = 98.486 (8)°
*V* = 3298.5 (19) Å^3^
*Z* = 4
Mo *K*α radiation
μ = 0.57 mm^−1^
*T* = 100 K
0.34 × 0.32 × 0.30 mm

#### Data collection

Bruker SMART CCD area-detector diffractometer
Absorption correction: multi-scan (*SADABS*; Bruker, 2001 ▶) *T*_min_ = 0.830, *T*_max_ = 0.848
18829 measured reflections
6796 independent reflections
3770 reflections with *I* > 2σ(*I*)
*R*_int_ = 0.111

#### Refinement

*R*[*F* ^2^ > 2σ(*F* ^2^)] = 0.069
*wR*(*F* ^2^) = 0.184
*S* = 0.97
6796 reflections
432 parameters
H atoms treated by a mixture of independent and constrained refinement
Δρ_max_ = 0.80 e Å^−3^
Δρ_min_ = −0.45 e Å^−3^

### 

Data collection: *SMART* (Bruker, 2001 ▶); cell refinement: *SAINT* (Bruker, 2001 ▶); data reduction: *SAINT*; program(s) used to solve structure: *SHELXS97* (Sheldrick, 2008 ▶); program(s) used to refine structure: *SHELXL97* (Sheldrick, 2008 ▶); molecular graphics: *SHELXTL* (Sheldrick, 2008 ▶); software used to prepare material for publication: *DIAMOND* (Brandenburg, 1999 ▶).

## Supplementary Material

Crystal structure: contains datablock(s) I, global. DOI: 10.1107/S1600536812000153/bt5764sup1.cif

Structure factors: contains datablock(s) I. DOI: 10.1107/S1600536812000153/bt5764Isup2.hkl

Additional supplementary materials:  crystallographic information; 3D view; checkCIF report

## Figures and Tables

**Table 1:** Hydrogen-bond geometry (Å, °)

*D*—H⋯*A*	*D*—H	H⋯*A*	*D*⋯*A*	*D*—H⋯*A*
O2—H01⋯O4	1.18 (6)	1.30 (6)	2.480 (4)	178 (9)
O1—H02⋯O3	1.18 (6)	1.27 (6)	2.446 (4)	173 (5)

**Fig. 1.**
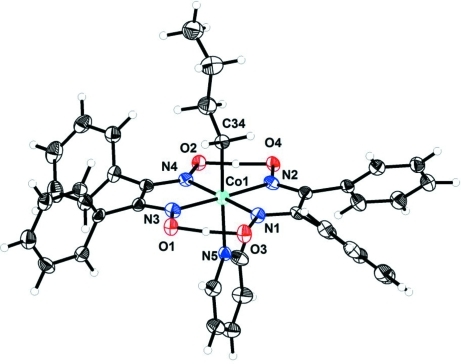
ORTEP diagram of the title compound is drawn of the 50% probability level for non-hydrogen atoms.

**Fig. 2.**
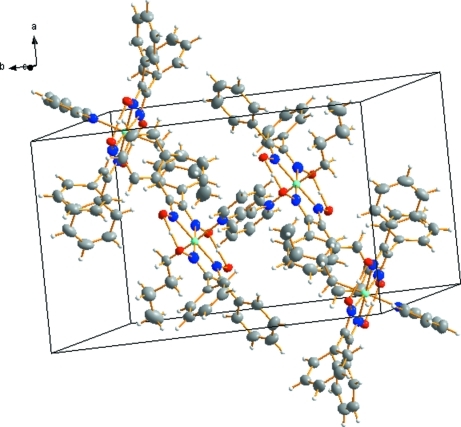
Packing of the complex in the unit cell.

## supplementary crystallographic information

### Comment

One of the unique and intriguing properties of the coenzyme B_12_ is different catalytic activity of two different coenzymes. How the Co—C bond is activated toward homolysis or heterolysis is an enduring subject of research (Zangrando *et al.*, 2003; Brown, 2006; Randaccio, 1999). The recent crystallographic data on cobalamins suggest that the structural effects of changes in the alkyl residue are similar (Kumar & Gupta, 2011) to those found in the cobaloximes, RCo(dmgH)_2_B, and sometimes can be related to their chemical reactivity (Mandal & Gupta, 2005, 2007). Cobaloximes have the general formula RCo(*L*)_2_B, where *R* is an organic group bonded to cobalt. B is an axial base *trans* to the organic group, and *L* is a monoanionic dioxime ligand. Diphenylglyoximate (dpg) is a familiar ligand with excellent coordination capability to generate mono-, bi- or trinuclear complexes. Herein, we report the synthesis and structure of new cobaloxime compound, namely butyl-Co(dpgH)_2_pyridine.

The crystal structure of title compound is shown in Figure 1. The cobalt atom is coordinated by two N,N-bidentate diphenylglyoximate ligands, a butyl and a pyridyl residue in a distored octahedral geometry. The Co—N(dpg) bond lengths range from 1.877 (3) to 1.886 (3) Å.

### Experimental

A solution of ClCo(dpgH)_2_py (1 mmol) in 10 ml of methanol was purged thoroughly with N_2_ for 20 min and was cooled to 0 °C with stirring. The solution turned deep blue after the addition of a few drops of aqueous NaOH followed by sodium borohydride (1.5 mmol in 0.5 ml of water). The color of the solution turned orange-red on the addition of bromobutane (1.5 mmol). The reaction was stirred 1 h at 0 °C then poured into 20 ml chilled water. The resulting orange-red precipitate was filtered, washed with water, and dried. The obtained orange colored compound was recrytallized from dichloromethane and methanol. After five days, orange colored crystals obtained which were suitable for single-crystal data collection.

### Refinement

H atoms were visible in difference Fourier maps but those bonded to C atoms were placed idealized positions and included in the refinement in a riding-model approximation with C—H(aromatic) = 0.95 Å and *U*_iso_(H) = 1.2 *U*_eq_C; C—H(methylene) = 0.99 Å and *U*_iso_(H) = 1.2 *U*_eq_C; C—H (methyl) = 0.99 Å, and *U*_iso_(H) = 1.5 *U*_eq_C. H atoms bonded to O atoms in the compound were refined freely with isotropic displacement parameters.

### Crystal data

**Table:**

[Co(C_4_H_9_)(C_14_H_11_N_2_O_2_)_2_(C_5_H_5_N)]	*F*(000) = 1408
*M**_r_* = 673.64	*D*_x_ = 1.357 Mg m^−^^3^
Monoclinic, *P*2_1_/*n*	Mo *K*α radiation, λ = 0.71073 Å
Hall symbol: -P 2yn	Cell parameters from 1034 reflections
*a* = 11.863 (4) Å	θ = 2.3–18.6°
*b* = 19.520 (7) Å	µ = 0.57 mm^−^^1^
*c* = 14.402 (5) Å	*T* = 100 K
β = 98.486 (8)°	Prism, orange
*V* = 3298.5 (19) Å^3^	0.34 × 0.32 × 0.30 mm
*Z* = 4	

### Data collection

**Table:**

Bruker SMART CCD area-detector diffractometer	6796 independent reflections
Radiation source: fine-focus sealed tube	3770 reflections with *I* > 2σ(*I*)
graphite	*R*_int_ = 0.111
phi and ω scans	θ_max_ = 26.5°, θ_min_ = 2.0°
Absorption correction: multi-scan (*SADABS*; Bruker, 2001)	*h* = −14→13
*T*_min_ = 0.830, *T*_max_ = 0.848	*k* = −16→24
18829 measured reflections	*l* = −18→18

### Refinement

**Table:**

Refinement on *F*^2^	Primary atom site location: structure-invariant direct methods
Least-squares matrix: full	Secondary atom site location: difference Fourier map
*R*[*F*^2^ > 2σ(*F*^2^)] = 0.069	Hydrogen site location: inferred from neighbouring sites
*wR*(*F*^2^) = 0.184	H atoms treated by a mixture of independent and constrained refinement
*S* = 0.97	*w* = 1/[σ^2^(*F*_o_^2^) + (0.0751*P*)^2^] where *P* = (*F*_o_^2^ + 2*F*_c_^2^)/3
6796 reflections	(Δ/σ)_max_ = 0.001
432 parameters	Δρ_max_ = 0.80 e Å^−^^3^
0 restraints	Δρ_min_ = −0.45 e Å^−^^3^

### Special details

**Table:**

Geometry. All s.u.'s (except the s.u. in the dihedral angle between two l.s. planes) are estimated using the full covariance matrix. The cell s.u.'s are taken into account individually in the estimation of s.u.'s in distances, angles and torsion angles; correlations between s.u.'s in cell parameters are only used when they are defined by crystal symmetry. An approximate (isotropic) treatment of cell s.u.'s is used for estimating s.u.'s involving l.s. planes.
Refinement. Refinement of *F*^2^ against ALL reflections. The weighted *R*-factor *wR* and goodness of fit *S* are based on *F*^2^, conventional *R*-factors *R* are based on *F*, with *F* set to zero for negative *F*^2^. The threshold expression of *F*^2^ > 2σ(*F*^2^) is used only for calculating *R*-factors(gt) *etc*. and is not relevant to the choice of reflections for refinement. *R*-factors based on *F*^2^ are statistically about twice as large as those based on *F*, and *R*- factors based on ALL data will be even larger.

### Fractional atomic coordinates and isotropic or equivalent isotropic displacement parameters (Å^2^)

**Table:**

	*x*	*y*	*z*	*U*_iso_*/*U*_eq_	
Co1	0.08170 (5)	0.10089 (3)	0.75782 (4)	0.02066 (19)	
O1	0.0991 (2)	0.10204 (16)	0.56369 (19)	0.0229 (7)	
O4	0.0709 (2)	0.10561 (15)	0.95271 (19)	0.0218 (7)	
O3	−0.0756 (3)	0.15157 (16)	0.6064 (2)	0.0262 (8)	
O2	0.2395 (2)	0.04611 (16)	0.90772 (19)	0.0224 (7)	
C16	−0.0827 (4)	0.1505 (2)	0.8539 (3)	0.0193 (10)	
N1	−0.0509 (3)	0.14466 (19)	0.7007 (2)	0.0208 (8)	
N2	0.0184 (3)	0.12110 (18)	0.8665 (2)	0.0204 (8)	
N3	0.1476 (3)	0.08265 (19)	0.6496 (2)	0.0226 (9)	
C23	−0.1499 (4)	0.1671 (2)	0.9301 (3)	0.0195 (10)	
C32	−0.1087 (4)	−0.0783 (2)	0.6532 (3)	0.0301 (11)	
H32	−0.1412	−0.0948	0.5931	0.036*	
N5	−0.0019 (3)	0.00897 (18)	0.7464 (2)	0.0204 (8)	
C7	0.3130 (4)	−0.0451 (3)	0.4455 (3)	0.0300 (12)	
H7	0.2973	−0.0879	0.4147	0.036*	
C18	−0.2561 (4)	0.2355 (2)	0.6420 (3)	0.0261 (11)	
H18	−0.1915	0.2543	0.6193	0.031*	
C9	0.3916 (4)	−0.0057 (2)	0.7914 (3)	0.0218 (10)	
C2	0.2842 (4)	0.0320 (2)	0.7575 (3)	0.0197 (10)	
N4	0.2157 (3)	0.05638 (18)	0.8134 (2)	0.0191 (8)	
C1	0.2425 (3)	0.0475 (2)	0.6583 (3)	0.0200 (10)	
C28	−0.1957 (4)	0.2316 (2)	0.9378 (3)	0.0250 (11)	
H28	−0.1777	0.2673	0.8976	0.030*	
C33	−0.0484 (4)	−0.0177 (2)	0.6624 (3)	0.0264 (11)	
H33	−0.0390	0.0067	0.6070	0.032*	
C4	0.3626 (4)	0.0782 (2)	0.5362 (3)	0.0231 (10)	
H4	0.3804	0.1206	0.5671	0.028*	
C13	0.5124 (4)	−0.1048 (3)	0.7867 (4)	0.0379 (13)	
H13	0.5258	−0.1485	0.7615	0.046*	
C21	−0.4455 (4)	0.1830 (3)	0.7107 (3)	0.0294 (11)	
H21	−0.5104	0.1657	0.7345	0.035*	
C15	−0.1253 (4)	0.1635 (2)	0.7544 (3)	0.0184 (9)	
C3	0.2963 (3)	0.0297 (2)	0.5754 (3)	0.0213 (10)	
C34	0.1579 (4)	0.1948 (2)	0.7681 (3)	0.0216 (10)	
H34A	0.1735	0.2080	0.7048	0.026*	
H34B	0.1025	0.2283	0.7864	0.026*	
C22	−0.3364 (4)	0.1631 (2)	0.7510 (3)	0.0232 (10)	
H22	−0.3268	0.1317	0.8020	0.028*	
C14	0.4128 (4)	−0.0689 (2)	0.7534 (3)	0.0262 (11)	
H14	0.3593	−0.0878	0.7045	0.031*	
C27	−0.2677 (4)	0.2452 (3)	1.0034 (3)	0.0286 (11)	
H27	−0.2972	0.2900	1.0094	0.034*	
C8	0.2713 (4)	−0.0323 (2)	0.5285 (3)	0.0247 (10)	
H8	0.2257	−0.0656	0.5536	0.030*	
C24	−0.1761 (4)	0.1151 (2)	0.9898 (3)	0.0272 (11)	
H24	−0.1443	0.0707	0.9858	0.033*	
C17	−0.2410 (4)	0.1892 (2)	0.7163 (3)	0.0232 (10)	
C25	−0.2482 (4)	0.1279 (3)	1.0547 (3)	0.0323 (12)	
H25	−0.2648	0.0924	1.0957	0.039*	
C19	−0.3644 (4)	0.2543 (2)	0.6010 (3)	0.0290 (11)	
H19	−0.3742	0.2849	0.5492	0.035*	
C10	0.4719 (4)	0.0221 (3)	0.8616 (3)	0.0282 (11)	
H10	0.4585	0.0655	0.8879	0.034*	
C11	0.5716 (4)	−0.0135 (3)	0.8933 (3)	0.0341 (12)	
H11	0.6264	0.0058	0.9409	0.041*	
C5	0.4023 (4)	0.0643 (3)	0.4527 (3)	0.0284 (11)	
H5	0.4474	0.0974	0.4269	0.034*	
C6	0.3776 (4)	0.0035 (3)	0.4064 (3)	0.0334 (12)	
H6	0.4043	−0.0053	0.3485	0.040*	
C20	−0.4589 (4)	0.2282 (3)	0.6357 (3)	0.0345 (13)	
H20	−0.5332	0.2415	0.6078	0.041*	
C26	−0.2961 (4)	0.1916 (3)	1.0605 (3)	0.0352 (13)	
H26	−0.3485	0.1993	1.1033	0.042*	
C30	−0.0729 (4)	−0.0892 (3)	0.8195 (3)	0.0331 (12)	
H30	−0.0796	−0.1136	0.8755	0.040*	
C35	0.2666 (4)	0.2021 (3)	0.8350 (4)	0.0382 (13)	
H35A	0.3256	0.1726	0.8138	0.046*	
H35B	0.2539	0.1859	0.8978	0.046*	
C36	0.3102 (5)	0.2757 (3)	0.8432 (4)	0.0480 (15)	
H36A	0.3158	0.2936	0.7797	0.058*	
H36B	0.2549	0.3045	0.8707	0.058*	
C31	−0.1206 (4)	−0.1145 (3)	0.7337 (3)	0.0322 (12)	
H31	−0.1614	−0.1565	0.7297	0.039*	
C12	0.5915 (4)	−0.0767 (3)	0.8561 (4)	0.0383 (13)	
H12	0.6596	−0.1008	0.8783	0.046*	
C29	−0.0152 (4)	−0.0279 (2)	0.8232 (3)	0.0297 (11)	
H29	0.0169	−0.0106	0.8830	0.036*	
C37	0.4250 (5)	0.2807 (3)	0.9033 (4)	0.0560 (17)	
H37A	0.4501	0.3286	0.9068	0.084*	
H37B	0.4803	0.2529	0.8757	0.084*	
H37C	0.4195	0.2639	0.9666	0.084*	
H01	0.158 (5)	0.074 (3)	0.928 (4)	0.062 (17)*	
H02	0.012 (5)	0.126 (3)	0.579 (4)	0.08 (2)*	

### Atomic displacement parameters (Å^2^)

**Table:**

	*U*^11^	*U*^22^	*U*^33^	*U*^12^	*U*^13^	*U*^23^
Co1	0.0225 (3)	0.0236 (4)	0.0154 (3)	0.0019 (3)	0.0012 (2)	0.0017 (3)
O1	0.0239 (17)	0.0320 (19)	0.0124 (15)	0.0063 (15)	0.0015 (13)	0.0050 (14)
O4	0.0236 (17)	0.0301 (18)	0.0109 (15)	0.0036 (14)	0.0000 (12)	−0.0029 (13)
O3	0.0278 (18)	0.035 (2)	0.0155 (16)	0.0070 (15)	0.0038 (13)	0.0037 (14)
O2	0.0199 (16)	0.0324 (19)	0.0136 (16)	0.0033 (14)	−0.0014 (12)	0.0001 (13)
C16	0.021 (2)	0.019 (2)	0.017 (2)	0.0008 (19)	0.0005 (18)	−0.0004 (18)
N1	0.022 (2)	0.026 (2)	0.0141 (19)	0.0007 (17)	0.0026 (15)	0.0036 (16)
N2	0.028 (2)	0.022 (2)	0.0099 (18)	0.0007 (17)	−0.0005 (15)	0.0001 (15)
N3	0.029 (2)	0.027 (2)	0.0115 (19)	−0.0001 (18)	0.0014 (16)	0.0019 (16)
C23	0.021 (2)	0.024 (3)	0.012 (2)	0.0025 (19)	−0.0016 (17)	−0.0025 (18)
C32	0.038 (3)	0.032 (3)	0.018 (3)	0.001 (2)	−0.004 (2)	−0.001 (2)
N5	0.020 (2)	0.025 (2)	0.0164 (19)	0.0017 (17)	0.0031 (15)	−0.0013 (16)
C7	0.034 (3)	0.030 (3)	0.024 (3)	0.006 (2)	−0.003 (2)	−0.009 (2)
C18	0.031 (3)	0.024 (3)	0.023 (3)	0.002 (2)	0.005 (2)	0.003 (2)
C9	0.020 (2)	0.029 (3)	0.016 (2)	0.004 (2)	0.0034 (18)	0.0070 (19)
C2	0.022 (2)	0.020 (2)	0.017 (2)	−0.0015 (19)	0.0023 (18)	−0.0005 (18)
N4	0.022 (2)	0.025 (2)	0.0093 (18)	−0.0008 (17)	−0.0005 (15)	0.0019 (15)
C1	0.016 (2)	0.022 (2)	0.021 (2)	0.0009 (19)	0.0014 (18)	0.0004 (19)
C28	0.026 (3)	0.027 (3)	0.020 (2)	0.002 (2)	−0.001 (2)	0.002 (2)
C33	0.027 (3)	0.035 (3)	0.017 (2)	0.001 (2)	−0.0003 (19)	0.004 (2)
C4	0.022 (2)	0.027 (3)	0.020 (2)	0.000 (2)	0.0009 (19)	−0.002 (2)
C13	0.040 (3)	0.038 (3)	0.037 (3)	0.016 (3)	0.010 (2)	0.005 (3)
C21	0.025 (3)	0.040 (3)	0.023 (3)	−0.001 (2)	0.002 (2)	−0.005 (2)
C15	0.026 (2)	0.015 (2)	0.014 (2)	0.0001 (19)	0.0034 (18)	−0.0002 (18)
C3	0.012 (2)	0.028 (3)	0.023 (2)	0.0008 (19)	−0.0004 (18)	−0.001 (2)
C34	0.023 (2)	0.022 (2)	0.020 (2)	−0.002 (2)	0.0026 (19)	0.0026 (19)
C22	0.024 (2)	0.030 (3)	0.014 (2)	0.003 (2)	−0.0010 (18)	0.000 (2)
C14	0.026 (3)	0.033 (3)	0.021 (2)	0.001 (2)	0.005 (2)	0.003 (2)
C27	0.025 (3)	0.033 (3)	0.027 (3)	0.004 (2)	0.000 (2)	−0.007 (2)
C8	0.021 (2)	0.031 (3)	0.022 (2)	0.000 (2)	0.0032 (19)	0.002 (2)
C24	0.036 (3)	0.024 (3)	0.022 (3)	0.005 (2)	0.004 (2)	0.001 (2)
C17	0.028 (3)	0.023 (3)	0.017 (2)	0.003 (2)	−0.0015 (19)	−0.0023 (19)
C25	0.041 (3)	0.031 (3)	0.028 (3)	−0.003 (2)	0.015 (2)	0.002 (2)
C19	0.040 (3)	0.023 (3)	0.023 (3)	0.005 (2)	0.002 (2)	−0.002 (2)
C10	0.028 (3)	0.032 (3)	0.025 (3)	0.002 (2)	0.003 (2)	0.000 (2)
C11	0.022 (3)	0.049 (4)	0.029 (3)	0.001 (2)	−0.004 (2)	0.006 (2)
C5	0.025 (3)	0.036 (3)	0.025 (3)	0.002 (2)	0.007 (2)	0.003 (2)
C6	0.030 (3)	0.047 (3)	0.022 (3)	0.008 (3)	0.001 (2)	−0.001 (2)
C20	0.029 (3)	0.043 (3)	0.027 (3)	0.010 (2)	−0.010 (2)	−0.004 (2)
C26	0.036 (3)	0.040 (3)	0.032 (3)	−0.002 (3)	0.012 (2)	−0.013 (2)
C30	0.045 (3)	0.032 (3)	0.021 (3)	−0.007 (3)	0.002 (2)	0.005 (2)
C35	0.040 (3)	0.032 (3)	0.044 (3)	−0.002 (3)	0.008 (3)	−0.002 (3)
C36	0.047 (4)	0.047 (4)	0.048 (4)	−0.007 (3)	−0.001 (3)	0.000 (3)
C31	0.039 (3)	0.029 (3)	0.028 (3)	−0.007 (2)	0.003 (2)	−0.001 (2)
C12	0.028 (3)	0.055 (4)	0.032 (3)	0.013 (3)	0.002 (2)	0.011 (3)
C29	0.040 (3)	0.029 (3)	0.018 (2)	−0.001 (2)	−0.002 (2)	−0.003 (2)
C37	0.056 (4)	0.065 (4)	0.051 (4)	−0.022 (3)	0.021 (3)	−0.018 (3)

### Geometric parameters (Å, °)

**Table:**

Co1—N1	1.870 (4)	C13—H13	0.9500
Co1—N2	1.875 (3)	C21—C20	1.384 (7)
Co1—N3	1.879 (4)	C21—C22	1.394 (6)
Co1—N4	1.883 (3)	C21—H21	0.9500
Co1—C34	2.039 (4)	C15—C17	1.488 (6)
Co1—N5	2.045 (4)	C3—C8	1.396 (6)
O1—N3	1.339 (4)	C34—C35	1.498 (6)
O1—H02	1.19 (7)	C34—H34A	0.9900
O4—N2	1.339 (4)	C34—H34B	0.9900
O4—H01	1.30 (6)	C22—C17	1.399 (6)
O3—N1	1.354 (4)	C22—H22	0.9500
O3—H02	1.27 (7)	C14—H14	0.9500
O2—N4	1.361 (4)	C27—C26	1.402 (7)
O2—H01	1.18 (6)	C27—H27	0.9500
C16—N2	1.318 (5)	C8—H8	0.9500
C16—C15	1.470 (6)	C24—C25	1.379 (6)
C16—C23	1.483 (6)	C24—H24	0.9500
N1—C15	1.308 (5)	C25—C26	1.375 (7)
N3—C1	1.308 (5)	C25—H25	0.9500
C23—C28	1.384 (6)	C19—C20	1.389 (7)
C23—C24	1.395 (6)	C19—H19	0.9500
C32—C33	1.378 (6)	C10—C11	1.389 (6)
C32—C31	1.383 (6)	C10—H10	0.9500
C32—H32	0.9500	C11—C12	1.378 (7)
N5—C29	1.348 (5)	C11—H11	0.9500
N5—C33	1.357 (5)	C5—C6	1.372 (7)
C7—C8	1.382 (6)	C5—H5	0.9500
C7—C6	1.390 (7)	C6—H6	0.9500
C7—H7	0.9500	C20—H20	0.9500
C18—C19	1.382 (6)	C26—H26	0.9500
C18—C17	1.391 (6)	C30—C31	1.373 (6)
C18—H18	0.9500	C30—C29	1.376 (6)
C9—C14	1.387 (6)	C30—H30	0.9500
C9—C10	1.393 (6)	C35—C36	1.525 (7)
C9—C2	1.489 (6)	C35—H35A	0.9900
C2—N4	1.315 (5)	C35—H35B	0.9900
C2—C1	1.472 (6)	C36—C37	1.506 (8)
C1—C3	1.476 (6)	C36—H36A	0.9900
C28—C27	1.389 (6)	C36—H36B	0.9900
C28—H28	0.9500	C31—H31	0.9500
C33—H33	0.9500	C12—H12	0.9500
C4—C5	1.380 (6)	C29—H29	0.9500
C4—C3	1.402 (6)	C37—H37A	0.9800
C4—H4	0.9500	C37—H37B	0.9800
C13—C12	1.380 (7)	C37—H37C	0.9800
C13—C14	1.397 (6)		
			
N1—Co1—N2	81.74 (15)	C35—C34—Co1	117.5 (3)
N1—Co1—N3	98.50 (15)	C35—C34—H34A	107.9
N2—Co1—N3	178.50 (16)	Co1—C34—H34A	107.9
N1—Co1—N4	179.07 (15)	C35—C34—H34B	107.9
N2—Co1—N4	99.17 (15)	Co1—C34—H34B	107.9
N3—Co1—N4	80.59 (15)	H34A—C34—H34B	107.2
N1—Co1—C34	87.49 (17)	C21—C22—C17	120.0 (4)
N2—Co1—C34	88.76 (17)	C21—C22—H22	120.0
N3—Co1—C34	89.78 (17)	C17—C22—H22	120.0
N4—Co1—C34	92.67 (16)	C9—C14—C13	120.3 (5)
N1—Co1—N5	89.95 (15)	C9—C14—H14	119.9
N2—Co1—N5	90.15 (15)	C13—C14—H14	119.9
N3—Co1—N5	91.33 (15)	C28—C27—C26	118.8 (5)
N4—Co1—N5	89.90 (15)	C28—C27—H27	120.6
C34—Co1—N5	177.33 (16)	C26—C27—H27	120.6
N3—O1—H02	102 (3)	C7—C8—C3	119.8 (4)
N2—O4—H01	97 (2)	C7—C8—H8	120.1
N1—O3—H02	102 (3)	C3—C8—H8	120.1
N4—O2—H01	97 (3)	C25—C24—C23	120.2 (4)
N2—C16—C15	112.5 (4)	C25—C24—H24	119.9
N2—C16—C23	124.8 (4)	C23—C24—H24	119.9
C15—C16—C23	122.6 (4)	C18—C17—C22	119.5 (4)
C15—N1—O3	120.0 (3)	C18—C17—C15	120.7 (4)
C15—N1—Co1	117.7 (3)	C22—C17—C15	119.7 (4)
O3—N1—Co1	122.0 (3)	C26—C25—C24	120.5 (5)
C16—N2—O4	121.0 (3)	C26—C25—H25	119.7
C16—N2—Co1	116.5 (3)	C24—C25—H25	119.7
O4—N2—Co1	122.5 (3)	C18—C19—C20	119.8 (4)
C1—N3—O1	118.9 (3)	C18—C19—H19	120.1
C1—N3—Co1	118.5 (3)	C20—C19—H19	120.1
O1—N3—Co1	122.5 (3)	C11—C10—C9	120.1 (5)
C28—C23—C24	119.1 (4)	C11—C10—H10	120.0
C28—C23—C16	121.2 (4)	C9—C10—H10	120.0
C24—C23—C16	119.4 (4)	C12—C11—C10	120.4 (5)
C33—C32—C31	118.3 (4)	C12—C11—H11	119.8
C33—C32—H32	120.9	C10—C11—H11	119.8
C31—C32—H32	120.9	C6—C5—C4	121.2 (5)
C29—N5—C33	116.4 (4)	C6—C5—H5	119.4
C29—N5—Co1	121.1 (3)	C4—C5—H5	119.4
C33—N5—Co1	122.5 (3)	C5—C6—C7	118.9 (4)
C8—C7—C6	121.1 (5)	C5—C6—H6	120.5
C8—C7—H7	119.5	C7—C6—H6	120.5
C6—C7—H7	119.5	C21—C20—C19	120.6 (5)
C19—C18—C17	120.5 (4)	C21—C20—H20	119.7
C19—C18—H18	119.8	C19—C20—H20	119.7
C17—C18—H18	119.8	C25—C26—C27	120.1 (4)
C14—C9—C10	119.2 (4)	C25—C26—H26	119.9
C14—C9—C2	120.3 (4)	C27—C26—H26	119.9
C10—C9—C2	120.4 (4)	C31—C30—C29	119.0 (4)
N4—C2—C1	111.7 (4)	C31—C30—H30	120.5
N4—C2—C9	123.7 (4)	C29—C30—H30	120.5
C1—C2—C9	124.7 (4)	C34—C35—C36	112.9 (4)
C2—N4—O2	120.1 (3)	C34—C35—H35A	109.0
C2—N4—Co1	117.8 (3)	C36—C35—H35A	109.0
O2—N4—Co1	122.0 (3)	C34—C35—H35B	109.0
N3—C1—C2	111.3 (4)	C36—C35—H35B	109.0
N3—C1—C3	120.9 (4)	H35A—C35—H35B	107.8
C2—C1—C3	127.8 (4)	C37—C36—C35	111.9 (5)
C23—C28—C27	121.1 (4)	C37—C36—H36A	109.2
C23—C28—H28	119.4	C35—C36—H36A	109.2
C27—C28—H28	119.4	C37—C36—H36B	109.2
N5—C33—C32	123.5 (4)	C35—C36—H36B	109.2
N5—C33—H33	118.2	H36A—C36—H36B	107.9
C32—C33—H33	118.2	C30—C31—C32	119.4 (5)
C5—C4—C3	120.1 (4)	C30—C31—H31	120.3
C5—C4—H4	119.9	C32—C31—H31	120.3
C3—C4—H4	119.9	C11—C12—C13	120.0 (5)
C12—C13—C14	119.9 (5)	C11—C12—H12	120.0
C12—C13—H13	120.0	C13—C12—H12	120.0
C14—C13—H13	120.0	N5—C29—C30	123.4 (4)
C20—C21—C22	119.7 (4)	N5—C29—H29	118.3
C20—C21—H21	120.2	C30—C29—H29	118.3
C22—C21—H21	120.2	C36—C37—H37A	109.5
N1—C15—C16	111.3 (4)	C36—C37—H37B	109.5
N1—C15—C17	122.9 (4)	H37A—C37—H37B	109.5
C16—C15—C17	125.6 (4)	C36—C37—H37C	109.5
C8—C3—C4	118.8 (4)	H37A—C37—H37C	109.5
C8—C3—C1	120.6 (4)	H37B—C37—H37C	109.5
C4—C3—C1	120.1 (4)		
			
N2—Co1—N1—C15	−4.5 (3)	Co1—N3—C1—C3	178.0 (3)
N3—Co1—N1—C15	177.0 (3)	N4—C2—C1—N3	0.7 (5)
N4—Co1—N1—C15	166 (45)	C9—C2—C1—N3	−178.4 (4)
C34—Co1—N1—C15	−93.6 (3)	N4—C2—C1—C3	179.1 (4)
N5—Co1—N1—C15	85.6 (3)	C9—C2—C1—C3	0.0 (7)
N2—Co1—N1—O3	−178.6 (3)	C24—C23—C28—C27	−0.5 (7)
N3—Co1—N1—O3	2.9 (3)	C16—C23—C28—C27	173.7 (4)
N4—Co1—N1—O3	−8(10)	C29—N5—C33—C32	1.3 (6)
C34—Co1—N1—O3	92.3 (3)	Co1—N5—C33—C32	−177.6 (4)
N5—Co1—N1—O3	−88.4 (3)	C31—C32—C33—N5	−1.2 (7)
C15—C16—N2—O4	179.8 (3)	O3—N1—C15—C16	178.9 (3)
C23—C16—N2—O4	−2.3 (6)	Co1—N1—C15—C16	4.8 (5)
C15—C16—N2—Co1	−1.5 (5)	O3—N1—C15—C17	3.4 (6)
C23—C16—N2—Co1	176.5 (3)	Co1—N1—C15—C17	−170.7 (3)
N1—Co1—N2—C16	3.2 (3)	N2—C16—C15—N1	−2.0 (5)
N3—Co1—N2—C16	103 (6)	C23—C16—C15—N1	179.9 (4)
N4—Co1—N2—C16	−176.7 (3)	N2—C16—C15—C17	173.3 (4)
C34—Co1—N2—C16	90.8 (3)	C23—C16—C15—C17	−4.7 (7)
N5—Co1—N2—C16	−86.8 (3)	C5—C4—C3—C8	−0.1 (6)
N1—Co1—N2—O4	−178.1 (3)	C5—C4—C3—C1	−172.7 (4)
N3—Co1—N2—O4	−79 (6)	N3—C1—C3—C8	−92.1 (6)
N4—Co1—N2—O4	2.0 (3)	C2—C1—C3—C8	89.6 (6)
C34—Co1—N2—O4	−90.5 (3)	N3—C1—C3—C4	80.4 (5)
N5—Co1—N2—O4	92.0 (3)	C2—C1—C3—C4	−97.9 (6)
N1—Co1—N3—C1	−176.0 (3)	N1—Co1—C34—C35	163.0 (4)
N2—Co1—N3—C1	85 (6)	N2—Co1—C34—C35	81.3 (4)
N4—Co1—N3—C1	3.8 (3)	N3—Co1—C34—C35	−98.4 (4)
C34—Co1—N3—C1	96.5 (3)	N4—Co1—C34—C35	−17.9 (4)
N5—Co1—N3—C1	−85.9 (3)	N5—Co1—C34—C35	147 (3)
N1—Co1—N3—O1	1.5 (3)	C20—C21—C22—C17	0.6 (7)
N2—Co1—N3—O1	−98 (6)	C10—C9—C14—C13	1.3 (6)
N4—Co1—N3—O1	−178.7 (3)	C2—C9—C14—C13	−178.7 (4)
C34—Co1—N3—O1	−85.9 (3)	C12—C13—C14—C9	−1.5 (7)
N5—Co1—N3—O1	91.6 (3)	C23—C28—C27—C26	−1.7 (7)
N2—C16—C23—C28	129.9 (5)	C6—C7—C8—C3	−1.5 (7)
C15—C16—C23—C28	−52.3 (6)	C4—C3—C8—C7	0.7 (6)
N2—C16—C23—C24	−56.0 (6)	C1—C3—C8—C7	173.3 (4)
C15—C16—C23—C24	121.8 (5)	C28—C23—C24—C25	0.9 (7)
N1—Co1—N5—C29	−118.8 (3)	C16—C23—C24—C25	−173.3 (4)
N2—Co1—N5—C29	−37.0 (3)	C19—C18—C17—C22	−1.7 (7)
N3—Co1—N5—C29	142.7 (3)	C19—C18—C17—C15	173.8 (4)
N4—Co1—N5—C29	62.1 (3)	C21—C22—C17—C18	0.5 (7)
C34—Co1—N5—C29	−103 (3)	C21—C22—C17—C15	−175.0 (4)
N1—Co1—N5—C33	60.1 (3)	N1—C15—C17—C18	−43.1 (6)
N2—Co1—N5—C33	141.8 (3)	C16—C15—C17—C18	142.0 (5)
N3—Co1—N5—C33	−38.4 (3)	N1—C15—C17—C22	132.3 (5)
N4—Co1—N5—C33	−119.0 (3)	C16—C15—C17—C22	−42.5 (6)
C34—Co1—N5—C33	76 (3)	C23—C24—C25—C26	0.8 (7)
C14—C9—C2—N4	128.6 (5)	C17—C18—C19—C20	1.7 (7)
C10—C9—C2—N4	−51.4 (6)	C14—C9—C10—C11	−0.4 (6)
C14—C9—C2—C1	−52.5 (6)	C2—C9—C10—C11	179.6 (4)
C10—C9—C2—C1	127.5 (5)	C9—C10—C11—C12	−0.4 (7)
C1—C2—N4—O2	179.0 (3)	C3—C4—C5—C6	0.2 (7)
C9—C2—N4—O2	−1.9 (6)	C4—C5—C6—C7	−1.0 (7)
C1—C2—N4—Co1	2.3 (5)	C8—C7—C6—C5	1.7 (7)
C9—C2—N4—Co1	−178.6 (3)	C22—C21—C20—C19	−0.6 (7)
N1—Co1—N4—C2	7(10)	C18—C19—C20—C21	−0.6 (7)
N2—Co1—N4—C2	178.2 (3)	C24—C25—C26—C27	−3.1 (7)
N3—Co1—N4—C2	−3.3 (3)	C28—C27—C26—C25	3.5 (7)
C34—Co1—N4—C2	−92.6 (3)	Co1—C34—C35—C36	−175.0 (4)
N5—Co1—N4—C2	88.1 (3)	C34—C35—C36—C37	−174.4 (5)
N1—Co1—N4—O2	−169 (45)	C29—C30—C31—C32	0.7 (7)
N2—Co1—N4—O2	1.6 (3)	C33—C32—C31—C30	0.2 (7)
N3—Co1—N4—O2	−179.9 (3)	C10—C11—C12—C13	0.3 (8)
C34—Co1—N4—O2	90.7 (3)	C14—C13—C12—C11	0.6 (8)
N5—Co1—N4—O2	−88.6 (3)	C33—N5—C29—C30	−0.4 (7)
O1—N3—C1—C2	178.9 (3)	Co1—N5—C29—C30	178.5 (4)
Co1—N3—C1—C2	−3.5 (5)	C31—C30—C29—N5	−0.6 (8)
O1—N3—C1—C3	0.4 (6)		

### Hydrogen-bond geometry (Å, °)

**Table:**

*D*—H···*A*	*D*—H	H···*A*	*D*···*A*	*D*—H···*A*
O2—H01···O4	1.18 (6)	1.30 (6)	2.480 (4)	178 (9)
O1—H02···O3	1.18 (6)	1.27 (6)	2.446 (4)	173 (5)
